# Reduced TRPC Channel Expression in Psoriatic Keratinocytes Is Associated with Impaired Differentiation and Enhanced Proliferation

**DOI:** 10.1371/journal.pone.0014716

**Published:** 2011-02-22

**Authors:** Kristina Leuner, Margarethe Kraus, Ute Woelfle, Heike Beschmann, Christian Harteneck, Wolf-Henning Boehncke, Christoph M. Schempp, Walter E. Müller

**Affiliations:** 1 Institute of Pharmacology, Biocenter Niederursel, Johann Wolfgang Goethe-University, Frankfurt, Germany; 2 Department of Dermatology, University Medical Center, Freiburg, Germany; 3 Department of Dermatology, Johann Wolfgang Goethe-University, Frankfurt, Germany; 4 Institute of Pharmacology and Toxicology & Interfaculty Centre for Pharmacogenomics and Drug Research, Eberhard-Karls-University, Tübingen, Germany; Hospital 12 Octubre Madrid, Spain

## Abstract

Psoriasis is a characteristic inflammatory and scaly skin condition with typical histopathological features including increased proliferation and hampered differentiation of keratinocytes. The activation of innate and adaptive inflammatory cellular immune responses is considered to be the main trigger factor of the epidermal changes in psoriatic skin. However, the molecular players that are involved in enhanced proliferation and impaired differentiation of psoriatic keratinocytes are only partly understood. One important factor that regulates differentiation on the cellular level is Ca^2+^. In normal epidermis, a Ca^2+^ gradient exists that is disturbed in psoriatic plaques, favoring impaired keratinocyte proliferation. Several TRPC channels such as TRPC1, TRPC4, or TRPC6 are key proteins in the regulation of high [Ca^2+^]_ex_ induced differentiation. Here, we investigated if TRPC channel function is impaired in psoriasis using calcium imaging, RT-PCR, western blot analysis and immunohistochemical staining of skin biopsies. We demonstrated substantial defects in Ca^2+^ influx in psoriatic keratinocytes in response to high extracellular Ca^2+^ levels, associated with a downregulation of all TRPC channels investigated, including TRPC6 channels. As TRPC6 channel activation can partially overcome this Ca^2+^ entry defect, specific TRPC channel activators may be potential new drug candidates for the topical treatment of psoriasis.

## Introduction

Psoriasis is a common chronic immune skin disease that affects more than 25 million people in North America and Europe [1]. It has a multifactorial etiology including cellular, genomic and genetic alterations, leading to microscopic and macroscopic disease-specific skin alterations, including inflammation, leukocyte infiltration and enhanced keratinocyte proliferation. Psoriatic lesions are characterized by thickened, irregular stratum corneum with parakeratosis and epidermal thickening with acanthosis and papillomatosis and absence of the granular layer. Thickening of the epidermis is a consequence of strongly increased keratinocyte proliferation, accompanied by reduced keratinocyte differentiation, eventually leading to reduced skin barrier function. These specific skin alterations are considered to be mainly the consequence of the inflammatory process and not a primary event in the pathophysiology [1], [2], [3]. The molecular mediators and intracellular signalling cascades of the inflammatory process involving T lymphocytes, dendritic cells and macrophages are well characterized [4]. However, the mediators which are responsible for enhanced proliferation and impaired differentiation of psoriatic keratinocytes are not identified yet. Proinflammatory cytokines such as IFN-γ, TGF-ß, IL-6 or IL-8 have no effect or even inhibit keratinocyte proliferation [5], [6]. The contribution of IL-20 and IL-22, cytokines produced by keratinocytes and T cells, to altered differentiation in psoriatic keratinocytes is currently discussed [7], [8]. However, a primary defect within the cellular mechanisms regulating keratinocyte maturation is also conceivable [9].

A first hint was reported nearly 20 years ago by Menon and Elias who described a defect of the epidermal calcium gradient in psoriatic skin [10]. In normal skin, extracellular Ca^2+^ ([Ca^2+^]_ex_) increases substantially from the basal over the spinous to the granular layer of the epidermis. As the calcium gradient is thought to represent an important physiological mechanism regulating keratinocyte maturation, defects in this system could easily explain altered proliferation and differentiation of psoriatic keratinocytes. However, the findings of Menon and Elias have so far not been reproduced by others.

Similar to the Ca^2+^ gradient in the epidermis, the differentiation and proliferation of isolated keratinocytes *in vitro* is regulated by an increase in intracellular Ca^2+^ ([Ca^2+^]_i_) via both Ca^2+^ release from intracellular stores and Ca^2+^ influx mechanisms. High extracellular Ca^2+^ concentrations ([Ca^2+^]_ex_) activate the Ca^2+^ sensing receptor (CaR), a G-protein-coupled receptor [11]. This leads to the stimulation of the phospholipase C (PLC) pathway, generating inositol 1,4,5-triphosphate (IP_3_) and diacylglycerol (DAG) [12]. IP_3_ as ligand of IP_3_ receptors induces release of Ca^2+^ from the endoplasmic reticulum (ER) and consecutive Ca^2+^ influx known as “store operated calcium influx” (SOCE). DAG directly activates members of the canonical transient receptor potential (TRPC) channel family and contributes to receptor operated calcium influx (ROC). Among the superfamily of TRP channels, TRPC channels have been suggested to be involved in SOCE, ROC and to play a key role in cell differentiation [13], [14].

TRPC channels can be divided into the TRPC1, TRPC4 and TRPC5 group and the TRPC3, TRPC6 and TRPC7 group, whereof DAG directly activates only the latter ones [15]. TRPC1 and TRPC4 seem to be involved in SOCE in keratinocytes, whereas TRPC6 and TRPC7 play an important role in ROC mediated Ca^2+^ influx [16], [17], [18], [19]. Furthermore, several TRPC channels are considered to be relevant for keratinocyte differentiation. TRPC1, TRPC4, as well as TRPC7 and TRPC6 have been implicated in the CaR triggered elevation of [Ca^2+^]_i_ and differentiation [20], [21], [19]. Importantly, we could show that specific activation of TRPC6 with hyperforin [22], [18] is sufficient to induce full differentiation and to inhibit proliferation similar to high [Ca^2+^]_ex_. In addition, TRPC1, TRPC5, TRPC6 and TRPC7 are upregulated after Ca^2+^-induced differentiation of gingival keratinocytes [21]. TRPC channels might also be involved in skin diseases such as Darier's disease [23] or actinic keratoses [24].

Therefore, we decided to test whether changes in [Ca^2+^]_i_ homeostasis including the CaR, the release of Ca^2+^ from the ER or TRPC channels are present in isolated psoriatic keratinocytes and are involved in the observed deficiencies in keratinocyte differentiation. We investigated primary keratinocytes isolated from psoriatic plaques with Ca^2+^ imaging, western blot analysis, RT-PCR and skin biopsies from psoriasis patients with immunohistochemical staining. We demonstrated substantial defects in Ca^2+^ influx in psoriatic keratinocytes in response to high extracellular Ca^2+^ levels, associated with a downregulation of all TRPC channels which are involved in ROC and SOCE, including TRPC6 channels which play a crucial role in keratinocyte differentiation. As TRPC6 channel activation could partially overcome this Ca^2+^ entry defect in psoriasis keratinocytes, specific TRPC6 channel activators e.g. out of the phloroglucinol class may be potential new drug candidates for the topical treatment of psoriasis.

## Results

### Impaired calcium homeostasis in psoriatic keratinocytes

**Figure 1:**
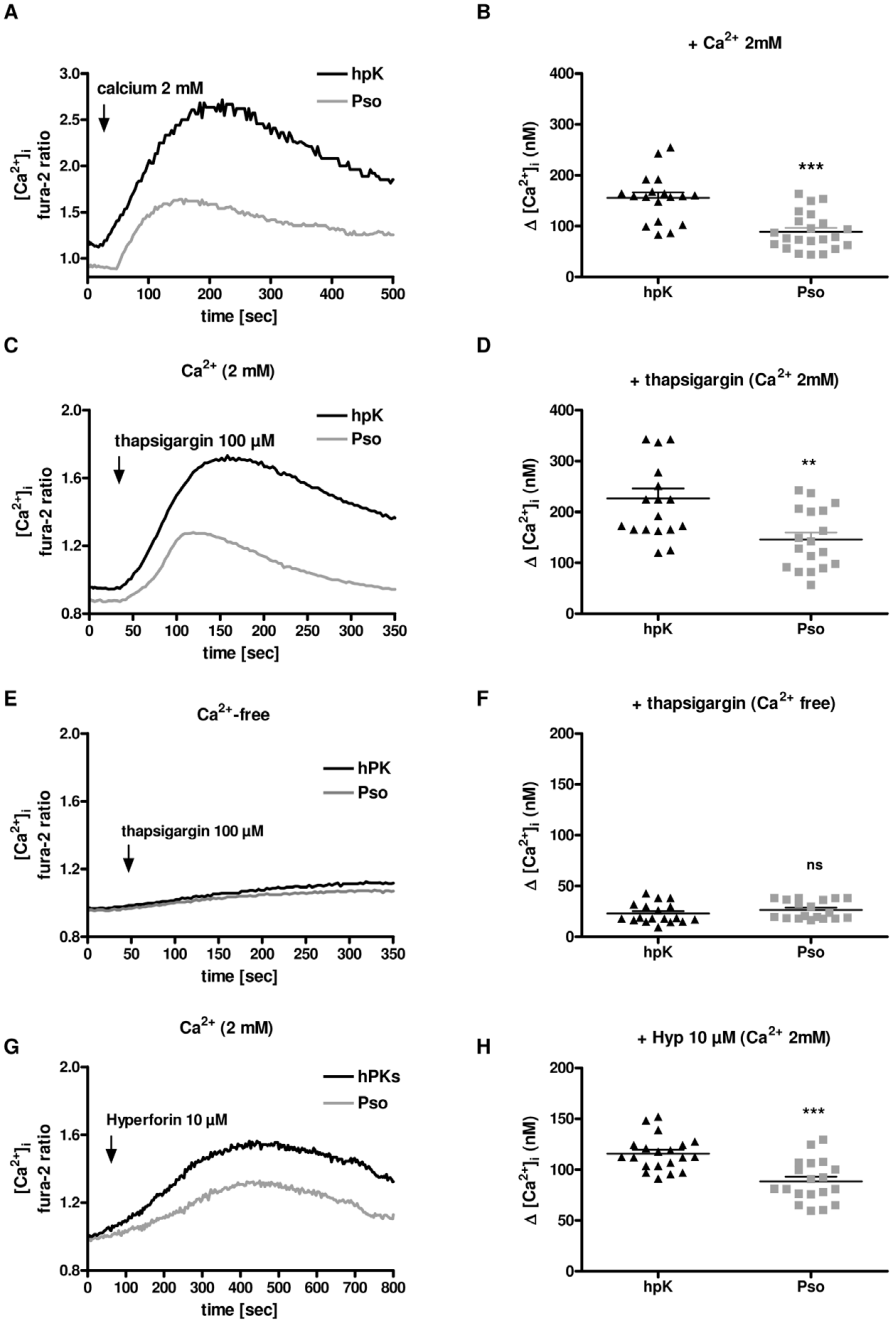
High [Ca^2+^]_ex_-, thapsigargin, and hyperforin-induced changes in [Ca^2+^]_i_ in psoriatic keratinocytes. Psoriatic keratinocytes and hpK were loaded with fura-2 and stimulated with high [Ca^2+^]_ex_ 2 mM, thapsigargin 100 µM, and hyperforin 10 µM (**A, B**). Representative time traces and mean changes in fura-2 ratio from high [Ca^2+^]_ex_-induced (2 mM) changes in [Ca^2+^]_i_ in psoriatic keratinocytes to hpK. Representative time traces show thapsigargin-induced elevation of [Ca^2+^]_i_ in calcium-containing buffer (**C**), and calcium-free buffer (**E**). Thapsigargin was added 50 s after the start of the experiment. **D and F,** the mean Ca^2+^-elevation in psoriatic keratinocytes compared to hpK after the stimulation with thapsigargin in buffer containing 1 mM Ca^2+^, or Ca^2+^-free buffer. **G and H,** hyperforin-induced calcium influx in psoriatic keratinocytes and hpK. Asterisks denote statistical significance as compared to hpK (psoriatic keratinocytes n = 6, 2 Ca^2+^ measurements per stimuli/patient; hpK n = 6, 2–4 Ca^2+^ measurements per stimuli/control;, *P<0.05, **P<0,01, ***P<0.001, ns = non significant unpaired *t* test).

To investigate if calcium homeostasis is impaired in psoriasis keratinocytes, we mimicked the steep calcium gradient in the skin using high [Ca^2+^]_ex_ (2 mM) and compared the increase in [Ca^2+^]_i_ of psoriatic keratinocytes and hpK. The resulting Ca^2+^ influx was strongly reduced in psoriatic keratinocytes (Fig. 1 A, B). We then asked if this defect is caused by the primary target of high [Ca^2+^]_ex_, the CaR, or consecutive players such as the release of Ca^2+^ from the ER or TRPC channels which are important in CaR mediated calcium influx. First experiments were conducted using thapsigargin in the presence and absence of [Ca^2+^]_ex_. Thapsigargin is an inhibitor of the endoplasmic reticulum (ER)-Ca^2+^ ATPase and was used to empty Ca^2+^ stores from ER [25]. The addition of thapsigargin (100 µM) in Ca^2+^-free medium raised slightly the [Ca^2+^]_i_ in both keratinocyte types (Fig. 1 E, F). Under these conditions, the increase of [Ca^2+^]_i_ is solely due to depletion of internal stores. No significant difference between the rise in [Ca^2+^]_i_ of psoriatic keratinocytes and hpK was observed.

In contrary to the results in Ca^2+^-free environment, in Ca^2+^-containing medium thapsigargin mobilizes not only a release from internal stores, but also a Ca^2+^-influx across the plasma membrane via store operated calcium channels (Fig. 1C, 1D). In fact, addition of thapsigargin (100 µM) in Ca^2+^-containing medium strongly increased the [Ca^2+^]_i_ in psoriatic keratinocytes and hpK (Fig. 1 C, D). However, the rise in [Ca^2+^]_i_ of psoriatic keratinocytes was significantly lower than the increase of [Ca^2+^]_i_ in hpK (Fig. 1D). This finding would be compatible with defects at the levels of ion channels mediating the store operated calcium influx such as TRPC1 or TRPC4 channels.

To test if TRPC channels, as downstream targets of CaR, might also be involved in impaired Ca^2+^ influx, the TRPC6 activator hyperforin [22], [18] was used to induce a pronounced Ca^2+^ influx. In psoriatic keratinocytes, [Ca^2+^]_i_ (Fig. 1G) was significantly decreased after hyperforin addition (Fig. 1H). These findings suggest that the CaR and/or TRPC channels might be involved in the defects in calcium entry in psoriatic keratinocytes. Importantly, basal Ca^2+^ levels were not different between psoriatric keratinocytes (79.1±11.2 nM) and hpK (61.2±8.8 nM).

### The CaR expression is reduced in lesional psoriatic keratinocytes

We investigated a possible role of CaR in the altered Ca^2+^ response to high [Ca^2+^]_ex_ by analyzing CaR mRNA levels in psoriatic keratinocytes isolated from 6 patients in comparison to 6 different hpK (Data S1). Keratinocytes from psoriatic lesions showed a slightly reduced expression of the receptor (20%) suggesting that reduced expression of CaR is not the major cause of the decreased Ca^2+^ influx in psoriatic keratinocytes. Furthermore, thapsigargin mediated Ca^2+^ influx does not differ between psoriatic keratinocytes and hpK. Therefore, we decided to further investigate the downstream targets of the signalling cascade, the TRPC channels.

### Involvement of TRPC channels in defects of psoriatic keratinocytes

**Figure 2:**
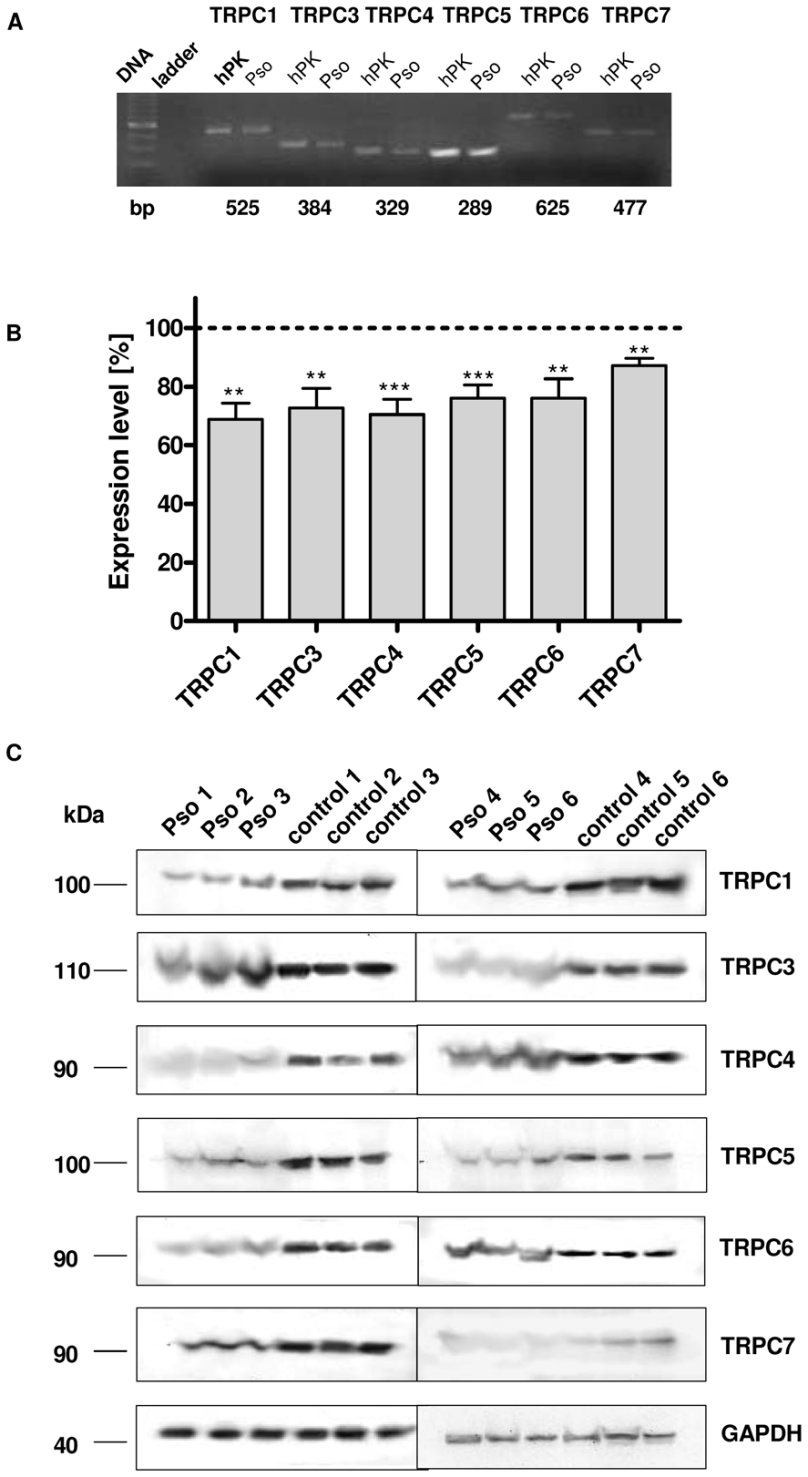
Reduced TRPC channel expression in psoriatic keratinocytes. **A,** mRNA expression of all TRPC channels in psoriatic keratinocytes and hpK was determined using RT-PCR analyses. **B,** Histogram reflecting relative expressing levels of the channels, compared to their normalized expression levels in hpK. TRPC levels of psoriasis patients were first normalized on their internal 18S control and then compared to hpK. Asterisks denote statistical significance as compared to control keratinocytes (*n* = 6, **P<0,01, ***P<0.001 unpaired *t* test). **C,** Western blotting of TRPC channels in protein samples taken stratum corneum from 6 psoriasis patients in comparison with stratum corneum obtained from 6 healthy volunteers. The protein levels have been normalized to GAPDH expression. Representative blots from single experiment that were repeated three times are shown.

**Figure 3:**
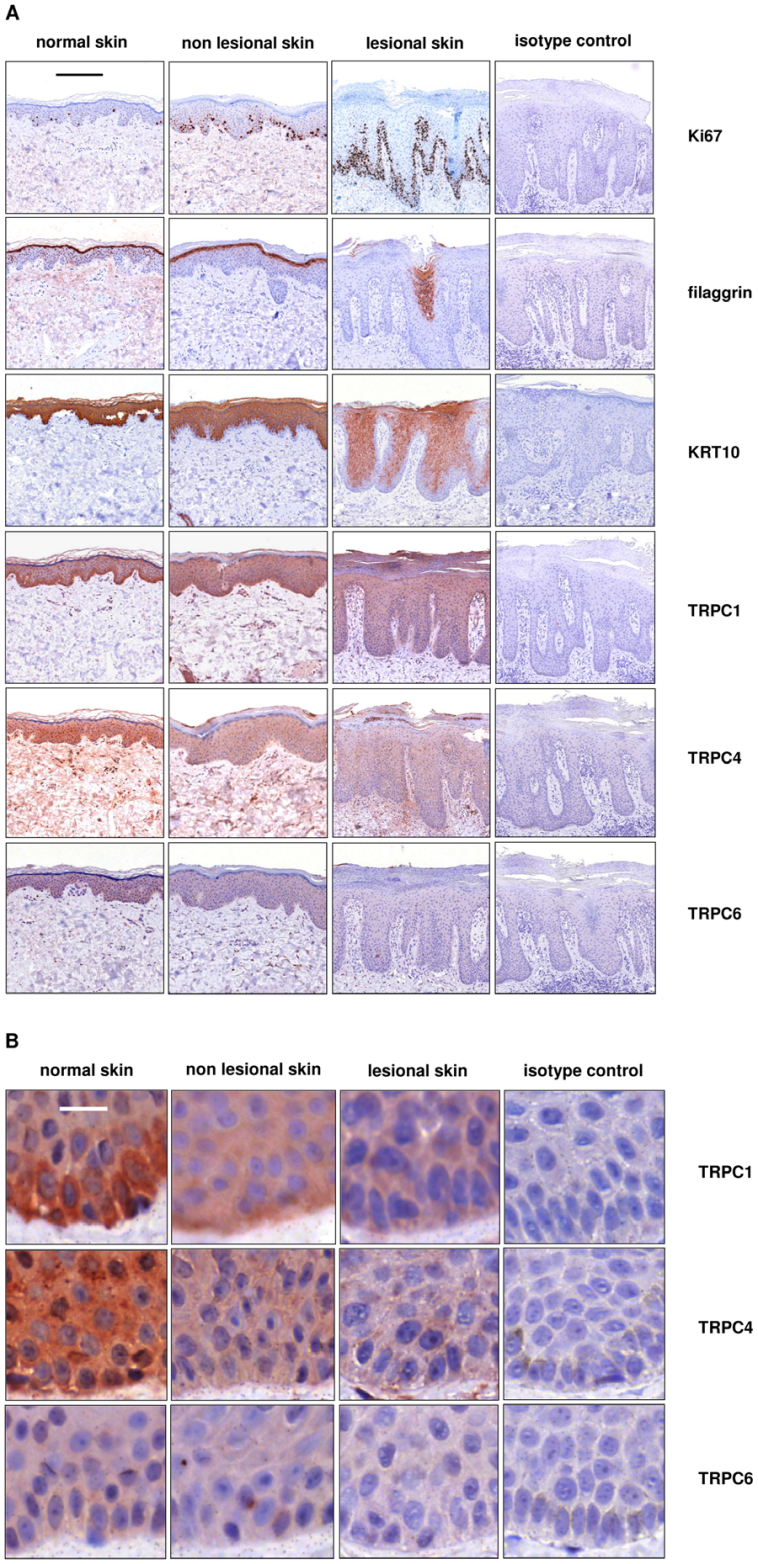
TRPC channel protein expression is reduced in immunohistochemical stainings of psoriatic lesional skin. Lesional and adjacent non lesional skin from psoriasis patients (n = 5) and skin from healthy controls (n = 6) were stained on the same slide for the expression of Ki67, filaggrin, KRT10, TRPC1, TRPC4 and TRPC6 using the LSAB method as described in the methods section. Isotype matched non-specific primary antibodies were used as negative controls. **A,** Histological scans showing staining patterns of the whole epidermis. Shown are representative sections (bar, 200 µm). **B,** Histological scans with a higher magnification showing more details of the celllular TRPC staining (bar, 10 µm).

To evaluate if the decreased receptor operated Ca^2+^ influx in psoriatic keratinocytes is caused by reduced expression levels of TRPC channels, we first performed RT-PCR analysis in keratinocytes isolated from psoriatic lesional skin (Fig. 2 A, B). As shown in Fig. 3 A, the mRNA levels of all members of the TRPC subfamily were significantly reduced in psoriatic keratinocytes compared to hpK. To investigate if TRPC channel protein levels are also down regulated in psoriatic skin, we obtained stratum corneum from psoriatic plaques of 6 patients and compared it to stratum corneum from 6 healthy controls. Western blot analysis of the plaques was performed to analyze the expression of TRPC channels (Fig. 2 C). The protein levels of the channels appear to be reduced in psoriatic plaques in comparison with proteins extracted out of stratum corneum from 6 volunteers. However, a correct quantification of the TRP protein bands was not possible because of the widening of the protein bands obtained for the psoriatic samples. This widending is suprising and currently not understood, it might be a reflection of degradation.

In addition, we performed immunohistochemical stainings on punch biopsies obtained from five lesional and five non lesional psoriasis skin samples compared to five healthy controls (Fig. 3). Firstly, the biopsies from untreated psoriasis lesions, non lesional skin from the same individuals and control skin were stained for the expression of the nuclear proliferation marker protein Ki67 that is expressed in cells undergoing the S/G2/M transition. Ki67 is a well established marker to detect proliferating cells [26]. As shown in Fig. 3 protein expression of Ki67 is strongly increased in lesional psoriasis epidermis and moderately increased in non lesional psoriasis skin, demonstrating the hyperproliferative condition of psoriatic keratinocytes. Secondly, we analyzed the expression of the late differentiation marker filaggrin. Filaggrin was only expressed in a patchy fashion in psoriasis lesions, whereas it formed a continuous layer at the transition from the stratum granulosum to stratum corneum in both non lesional psoriatic and normal skin (Fig. 3). Similarly the early differentiation marker K10 was analyzed. Compared to non lesional psoriatic skin and normal skin the number of K10 expressing keratinocyte layers was considerably lower in lesional psoriasis epidermis (Fig. 3), indicative of disturbed keratinocyte differentiation in psoriasis as it has already been demonstrated in several reports (for a comprehensive review please see Tschachler et al., 2007).

To explore if abnormalities in TRPC expression can also be demonstrated in non-lesional and lesional skin obtained from psoriasis patients using immunohistochemistry, we analyzed exemplary the expression of the TRPC1-, TRPC4- and TRPC6-channels which are known to be important players in CaR induced Ca^2+^ influx (Cai et al. 2006; Fatherazi et al., 2007; Müller et al., 2008). TRPC1 was mainly expressed in stratum basale of the epidermis in skin biopsies from healthy controls and, to a somewhat lesser extent in lesional and lesional psoriatic skin (Fig. 3). Similarly, the stainings for TRPC4 and TRPC6 were also weaker in psoriasis lesions (Fig. 3). The staining characteristics are summarized in Table 1.

**Table 1:** Characteristics of psoriasis patients (Pso) and control subjects (Con) and semiquantiative enumeration of immunohistological stainings.

Sample	Age	Sex	Localization	Ki67*	Filaggrin**	KRT10***	TRPC1****	TRPC4****	TRPC6****
Pso 1 lesional	67	F	Thigh	35/7	(+)	3	+ (focally ++)	+	(+)
Pso 2 lesional	49	F	Forearm	28/7	(+)	3	+	+ (focally −)	+ (diffuse)
Pso 3 lesional	40	M	Hip	24/5	(+)	2	+ (diffuse)	+	(+)
Pso 4 lesional	26	M	Forearm	28/8	(+)	4	+	+ (focally −)	+
Pso 5 lesional	57	F	Thigh	32/12	−	5	+	+	−
Pso 1 non lesional	67	F	Thigh	11/9	+	2	+ (basal ++)	+	(+)
Pso 2 non lesional	49	F	Forearm	15/6	+	2	++	++	(+)
Pso 3 non lesional	40	M	Hip	9/5	+	2	+ (basal ++)	++	(+)
Pso 4 non lesional	26	M	Forearm	11/9	+	1	++	++	(+)
Pso 5 non lesional	57	F	Thigh	15/8	+	2	+	+	(+)
Con 1	79	F	Back	4/2	+	2	++ (basal +++)	++	+
Con 2	74	F	Abdomen	10/4	+	2	+ (basal ++)	++	+
Con 3	63	M	Thigh	5/4	+	1	++	+++	+
Con 4	33	F	Thigh	6/3	+	1	++	++	(+)
Con 5	67	M	Thigh	2/1	+	2	++ (basal +++)	++	+

F, female; M, male.

*Ki67: number of positive cells/HPF (mean/SD).

**Filaggrin: staining intensity (− negative; (+) fragmentary; + continuous band in the upper epidermis).

***KRT10: number of unstained basal layers.

****TRPC1, TRPC4, TRPC6: staining intensity (− negative; (+) weak; + slightly positive; ++ marked positive; +++ strongly positive).

Taken together, our findings using RT-PCR, western blotting and immunohistochemistry strongly suggest down-regulated expression of TRPC channels in psoriasis on the mRNA and protein level.

### Disturbed differentiation and proliferation in psoriatic keratinocytes

**Figure 4:**
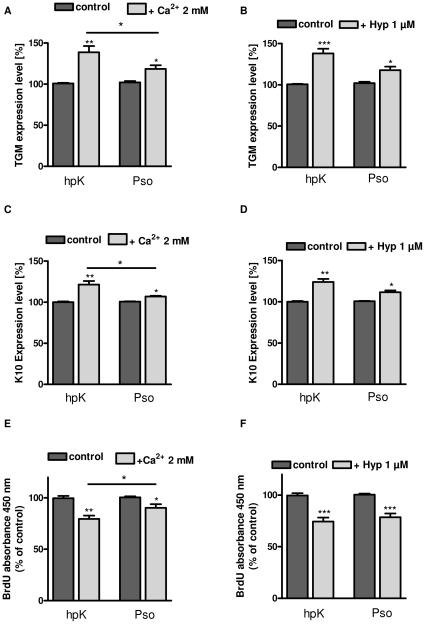
High [Ca^2+^]_ex_ and hyperforin-induced differentiation and proliferation of psoriatic keratinocytes. Psoriatic keratinocytes and hpK were incubated for 48 h with Ca^2+^ (2 mM, **A, C**) or hyperforin (1 µM, **B, D**). Total mRNA of treated cells was isolated, reverse transcribed and subjected to PCR. The expression of the differentiation markers K10 (**A, B**) and TGM I (**C, D**) were analyzed. Histograms are showing relative expression levels of the differentiation markers compared to their normalized expression levels in untreated controls. The baseline expression levels of TGM and K10 (normalized for the respective GAPDH expression) were somewhat different between patients and controls in these experiments carred out in the presence of [Ca^2+^]_ex_ 0.1 mM. TGM: hpK 0.54±0.13, PSO 0.38±0.19 (p = 0.11), K10 hpK 0.40±0.11, PSO 0.28±0.08 (p = 0.06). Asterisks denote statistical significance as compared to control keratinocytes (*n* = 5/6, *P<0.05, **P<0.01, ***P<0.001 unpaired *t* test). **E and F,** proliferation was detected using a BrdU immunoassay kit. Cells were incubated 48 h with Ca^2+^ (2 mM, E) or hyperforin (1 µM, F). Relative changes in percentage in the absorbance at 450 nm compared to the untreated control were measured (*n* = 6, *P<0.05, **P<0.01, ***P<0.001, unpaired *t* test).

Consequently, we asked if the decreased receptor operated calcium influx caused by decreased TRPC channel expression results in disturbed differentiation and proliferation of psoriatic keratinocytes. RT-PCR analyses revealed that hPK cells express basal levels of K10 and TGM1 which are up-regulated during high [Ca^2+^] treatment (Fig. 4A, C). K10 and TGM1 both are markers for keratinocyte differentiation. K10 and TGM1 levels were increased in psoriatic keratinocytes incubated with high [Ca^2+^]_ex_-containing medium (Fig. 4A, C), but to a much lower extent compared to hpK. This is also supported by the slightly reduced expression levels of both markers under baseline conditions (legend to Fig. 4). Our findings are compatible with the assumption of an impaired balance between differentiation and proliferation in psoriatic keratinocytes. Incubation of psoriasis keratinocytes and hpK with a high [Ca^2+^]_ex_ resulted in a reduced cell proliferation in both cell types. However, compared to hpK the anti-proliferative effect of high [Ca^2+^]_ex_ was significantly lower in psoriatic keratinocytes (Fig. 4 E).

In summary, these findings demonstrate that psoriatic keratinocytes display a disturbed balance between differentiation and proliferation, presumably due to a defect of the epidermal calcium homeostasis that is associated with a reduced expression of TRPC channels.

### TRPC6 activation partly restores the disturbed differentiation and proliferation in psoriatic keratinocytes

To investigate if increased TRPC channel function restores the abnormalities observed in psoriasis keratinocytes, the effect of the TRPC6 activator hyperforin (1 µM) on the expression of the differentiation markers K10 and TGM1 and on cell proliferation was investigated. Hyperforin significantly increased the expression of the early differentiation marker K10 and the late differentiation marker TGM1 in both psoriatic keratinocytes and hpK (Fig. 4 B, D). Even more important, hyperforin reduced the proliferation of hpK and psoriatic keratinocytes to similar extent (Fig. 4 F). While the inhibition of proliferation by hyperforin appears to be slightly higher than the effect of high [Ca^2+^]_ex_, its effects on the expression of the differentiation markers are rather similar. Taken together, these findings suggest that TRPC channel activation might be a promising treatment strategy to overcome the disturbed maturation of keratinocytes in psoriasis.

## Discussion

Intracellular calcium measurements, expression analysis and immunohistological staining allowed us to unravel the defects of psoriatic keratinocytes differentiation and the potential molecular elicitors. This study describes findings from *in vitro* a well as *ex vivo* analyses to clarify the pathomechanisms of keratinocyte dysfunction in psoriatic skin. Our data show that the impaired calcium homeostasis in psoriatic keratinocytes leading to defects in differentiation and proliferation is associated with downregulated expression of CaR and TRPC channels. Consequently, in calcium imaging experiments, psoriatic keratinocytes showed a reduced calcium influx after stimulation with high [Ca^2+^]_ex_. TRPC1, TRPC4, TRPC7 and TRPC6 have been implicated in the calcium-induced differentiation *in vitro* [21] although TRPC6 seems to play a substantial role, as its stimulation by its activator hyperforin is sufficient to induce a full differentiation response [18]. In agreement with the important role of TRPC6, the Ca^2+^ influx mediated by the TRPC6 activator hyperforin was significantly impaired in psoriatic keratinocytes. A reduced [Ca^2+^]_i_ in psoriasis keratinocytes has also been reported by Karvonen et al. (2000) following stimulation with thapsigargin, which has been explained by reduced Ca^2+^ release from the ER [27]. However, these experiments were conducted in the presence of high [Ca^2+^]_ex_. We were able to reproduce and to extend these findings indicating the primary role of defects in Ca^2+^ entry mechanisms such as SOCE as well as ROC.

As our results implicated a defect in CaR and/or TRPC channels, we examined the expression pattern of CaR and TRPC channels in cultured psoriatic keratinocytes compared to control human keratinocytes. By RT-PCR a slightly reduced expression of CaR could be detected which however seems to be too small to solely explain the pronounced changes in [Ca^2+^]_i_. In addition, the expression levels of all TRPC channels on mRNA and protein levels are significantly reduced both in cultured psoriatic keratinocytes and psoriasis plaques, as we could detect with RT-PCR and western blot analysis. Our findings of a diminished TRPC1-, TRPC4- and TRPC6-channel expression were confirmed by immunohistological stainings of punch biopsies from psoriasis plaques. This approach already demonstrated a downregulation of TRPC channels in non lesional skin which is even more pronounced in lesional skin from the same patient and healthy controls. This provides a more plausible explanation for the link between defects in calcium homeostasis and disturbed differentiation of psoriatic keratinocytes. Up to now, only external elicitors like a loss of the calcium gradient, abnormal barrier function or enhanced production of the cytokines IL-20 or IL-22 in T cells (and consecutively in keratinocytes) have been hypothesized to play a role in the altered differentiation and proliferation in psoriatic skin [10], [4]. Our findings show for the first time that a specific defect of calcium influx regulating mechanisms represent a major intrinsic feature of psoriasis keratinocytes, eventually leading to pronounced alterations in differentiation and proliferation. Importantly, changes in TRPC channel expression and defects in calcium signalling were also present in cultured psoriasis keratinocytes detached from their *in vivo* environment which is in agreement with many other studies (see the comprehensive review by Tschachler) [28]. Using a similar preparation, Wu et al. 2009 investigated the expression of filaggrin-2 in lesional and non-lesional skin from psoriasis patients and found only reduced expression in lesional skin [29]. Therefore, acute inflammatory processes and cytokines released by T cells cannot sufficiently explain the observed changes in calcium signalling. However, we cannot answer the question if decreased TRPC channel expression is acquired by inflammatory processes or if an intrinsic defect together with cytokines released from infiltrating T cells, contributes to keratinocyte alterations, resulting in acanthosis, hyperkeratosis and parakeratosis *in vivo*. Karvonen et al. (2000) postulated an inherited defect in calcium metabolism because the altered Ca^2+^ influx after store depletion was still present up to the fifth passage of cultured psoriatic keratinocytes and could be also detected in keratinocytes isolated from non lesional skin. These assumptions are supported by the findings that keratinocytes isolated from lesional and nonlesional skin equally appear to be “diseased” and that additional stressors such as cytokines can trigger the formation of psoriatic plaques [30], [8], [4], [7].

The hypothesis that TRPC channels might be a novel target for the treatment of psoriasis is supported by our findings that specific TRPC6 activation by hyperforin not only elevates [Ca^2+^]_i_ but also at least partially overcomes the intrinsic defect of maturation. Hyperforin increased keratinocyte differentiation as indicated by the enhanced expression of the differentiation markers K10 and TGM1 in psoriatic keratinocytes. However, hyperforin is not able to restore the defect completely to hpK control levels. Furthermore, hyperforin decreased proliferation in psoriasis keratinocytes and hpK to similar extent which might be another pathophysiologically important aspect. Furthermore, TRPC channels are discussed to be involved in Darier's disease and atopic dermatitis [23], [24]. However, therapeutical effectiveness of targeting TRPC channel function and expression as a potential therapeutical concept for psoriasis will require much more data. While hyperforin itself might be less useful due to its chemical instability, our findings that TRPC6 activation can be mimicked by rather simple phloroglucinol derivatives opens a further possibility to investigate this approach [31].

Taken together, our data shows for the first time that TRPC channels are involved in the pathogenesis of psoriasis and that they might be a promising new target for the treatment of psoriasis.

## Methods

### Sources and preparation of reagents

Hyperforin was a kind gift of Dr. Willmar Schwabe, Karlsruhe (Germany). Fluorescence dye fura-2-AM was purchased from Molecular Probes (Eugene, OR, U.S.A).

### Patients and skin samples

To obtain psoriatic keratinocytes, punch biopsies from typical psoriatic plaques of six patients were taken after written informed consent and the protocol was approved by the ethics committee of the University of Frankfurt, Germany. Skin biopsies for immunohistochemistry were from untreated psoriasis lesions and adjacent non lesional skin (n = 5) and from normal skin obtained from dermatological surgery (n = 5). All patients gave their written informed consent to use their skin in the study and the protocol was approved by the ethics committee of the University of Freiburg, Germany. The biopsies were fixed in paraformaldehyde, embedded in paraffin and stored at room temperature until immunohistochemical analysis.

### Cell culture

To isolate psoriatic keratinocytes, punch biopsies from typical psoriatic plaques of six patients were taken. Normal primary keratinocytes (hpK) were derived from adult skin obtained from dermatological surgery. Both types of keratinocytes were cultured according to the method of Rheinwald and Green [32] in keratinocyte growth medium (Promo Cell, Heidelberg, Germany) under a 5% CO_2_ humidified atmosphere at 37°C.

For cell culture experiments, keratinocytes were seeded in 6-well plates for RT-PCR and western blot, and on glass cover slips for Ca^2+^ imaging. For differentiation studies, cells were allowed to attach for 24 hours after trypsinization; subsequently 0.1 mM Ca^2+^-containing keratinocyte-SFM medium (Invitrogen) with 10% heat-inactivated fetal calf serum (Sigma-Aldrich). Afterwards, medium was replaced by SFM medium without fetal calf serum with 2 mM Ca^2+^ or 1 µM hyperforin. After 48 h incubation in the latter medium RT-PCR of corresponding markers was performed.

### Immunohistochemistry

3 µm sections of paraffin-embedded lesional and adjacent non lesional skin from psoriasis patients (n = 5) and skin from healthy controls (n = 5) were stained for Ki67, filaggrin, KRT10, TRPC1, TRPC4 and TRPC6 using the LSAB method (DCS, Hamburg, Germany) as described by [24]. The biopsies were stained with a specific antibody on the same slide. Isotype matched non-specific primary antibodies were used as negative controls.The following antibodies and dilutions were used: the primary polyclonal TRPC6 antibody (Chemicon, Schwalbach Germany), 1∶200; the polyclonal TRPC4 antibody (Santa Cruz Biotechnology, Inc., Heidelberg, Germany), 1∶50; the polyclonal TRPC1 antibody (Santa Cruz Biotechnology, Inc., Heidelberg, Germany), 1∶500; the polyclonal filaggrin antibody (Santa Cruz Biotechnology, Inc., Heidelberg, Germany), 1∶5000; the monoclonal KRT10 antibody (Santa Cruz Biotechnology, Inc., Heidelberg, Germany), 1∶1000; the polyclonal Ki-67 antibody (DCS, Hamburg, Germany), 1∶200; The matched isotype controls were rabbit IgG (Dako, Glostrup, Denmark, 1∶500), goat IgG (Dianova, 1∶200) and mouse IgG (Dako, 1∶1000). The secondary antibody multi-link-biotin, the streptavidin-HRP-label and the AEC-substrate were from Dako and were used according to the manufacturer's instruction. Stainings with isotype matched non specific primary antibodies served as control and were negative in all cases.

### RT-PCR

RNA was isolated using TRIzol reagent (Invitrogen, Karlsruhe, Germany), chloroform, and 100% ethanol according to manufacturer's instructions. Reactions were carried out using 2 µg of mRNA. First-strand cDNA was synthesized from 2 µg total RNA in 20 µl final volume using a First Strand cDNA Synthesis Kit (Invitrogen, Karlsruhe, Germany). After reverse transcription, amplification was carried out by PCR using the Taq DNA polymerase and dNTP Set of Invitrogen. A 2 µl aliquot of the reverse transcription solution was used as a template for specific PCR. The PCR primers used to amplify TRPC1, 3, 4, 5, 6 and 7 channels, TGM1, K10 and CaR cDNAs are specified in Table 2. Commercially available 18S rRNA primers (Ambion, Huntington, UK) were used as internal loading control and the predicted 18S (Classic II) band size was 324 bp. The PCR was conducted under the following conditions: an initial denaturation step at a temperature of 94°C for 5 min and 30 cycles as follows: 30 s at 94°C, 30 sec at 58°C, 30 sec at 72°C, and finally 7 min at 72°C. PCR products were run on a 1% agarose gel and stained with ethidium bromide. Changes in relative mRNA levels were obtained by relating each PCR product to its internal control. After gel electrophoresis quantification was archived with ChemiDoc XRS System and Quantity One Software (Bio-Rad).

**Table 2:** 

Name	Accession no.	Forward (5′→3′)	Backward (5′→3′)	Expected size
K10	NM_00421	GCAAAATCAAGGAGCGGTATGA	GAGCTGCACACAGTAGCGACC	685
TGMI	NM_000359	GATCGCATCACCCTTGAGTTAC	TCCTCATGGTCCACGTACACAAT	304
TRPC1	Z73903	ATGTATACAACCAGCTCTATCTTG	AGTCTTTGGTGAGGGAATGATG	525
TRPC3	U47050	CTGCAAATGAGAGCTTTGGC	AACTTCCATTCTACATCACTGTC	388
TRPC4	AF175406	ATTCATATACTGCCTTGTGTTG	GGTCAGCAATCAGTTGGTAAG	329
TRPC5	AF054568	ACTTCTATTATGAAACCAGAGC	GCATGATCGGCAATAAGCTG	289
TRPC6	AF080394	AAGACATCTTCAAGTTCATGGTC	TCAGCGTCATCCTCAATTTCC	322
TRPC7	AJ272034	GTCCGAATGCAAGGAAATCT	TGGGTTGTATTTGGCACCTC	477
CaR	U20760	ATGTGGTAGAGGTGATCCAA	GGCGTGGGCAATGGAGTAGA	529

### Western blotting

Keratinocytes from psoriasis plaques or normal epidermis from healthy controls were obtained by scratching off stratum corneum layers from psoriatic plaques or from heel stratum corneum respectively. The samples were transferred into PBS with complete® protease inhibitor cocktail from Roche Diagnostics, Germany and were used for Western blot experiments. Protein content was measured according to the Lowry method. Samples were mixed with Tris/glycine reducing buffer, denaturing loading buffer (both from Invitrogen™, Germany), loaded and electrophoresed on NuPAGE™ 4–12% Bis–Tris Gels (Invitrogen™, Germany). Gels were transferred to PVDF membranes (Amersham Biosciences), incubated overnight with the respective primary antibodies [TRPC1 (1/200): ACC -010 (Alomone, Israel); TRPC3 (1/200): ACC-016 (Alomone, Israel); TRPC4 (1/200): ACC-018 (Santa Cruz Biotechnology, Inc., Heidelberg, Germany); TRPC5 (1∶200) ACC-020: (Alomone, Israel); TRPC6 (1/200): ACC-017 (Alomone, Israel); TRPC7 (1∶200): AB9326 (Calbiochem, Germany)] and secondary antibodies (Calbiochem, Germany) conjugated to horseradish peroxidase and processed for visualization by ECL™ Reagent (Amersham Biosciences, Germany). The antibody for Glyceraldehyde-3-phosphate dehydrogenase (GAPDH, 1/300) (MAB374, Chemicon, Germany) served as loading control. Relative intensities of the bands were quantified by densitometry with ChemiDoc XRS System and Quantity One Software (Bio-Rad).

### Fluorescence measurements

The intracellular Ca^2+^ concentration [Ca^2+^]_i_, measurements in single cells were carried out using the fluorescence indicator fura-2-AM in combination with a monochromator-based imaging system (T.I.L.L. Photonics, Martinsried, Germany or Attofluor Ratio Vision system) attached to an inverted microscope (Axiovert 100, Carl Zeiss, Oberkochen, Germany). For [Ca^2+^]_i_ measurements psoriatic keratinocytes and hpK were loaded with 4 µM fura-2-AM (Invitrogen) and 0.01% Pluronic F-127 (Invitrogen) for 30 min at room temperature in a standard solution composed of 138 mM NaCl, 6 mM KCl, 1 mM MgCl_2_, 2 mM CaCl_2_, 5.5 mM glucose, and 10 mM HEPES (adjusted to pH 7.4 with NaOH). Cover slips were then washed in this buffer for 20 min and mounted in a perfusion chamber on the microscope stage. To measure [Ca^2+^]_i_ in Ca^2+^-free environment cells were loaded with fura-2-AM and then washed in Ca^2+^-free standard solution. For all fluorescence experiments, fluorescence was excited at 340 and 380 nm. After correction for background fluorescence, the fluorescence ratio *F*_340_/*F*_380_ was calculated.

### Proliferation measurement

Quantification of cell proliferation was determined by a non-isotopic immunoassay kit (Calbiochem, Germany), based on the measurement of BrdU incorporation during DNA synthesis. The assay was carried out according to the product instruction manual.

### Statistics

In addition to Microsoft Office Excel, GraphPad PRISM™ (Version 3.0) was used for statistical analyses and to create the graphs. For statistical analyses, an unpaired Student's *t*-test (two-tailed) was used. Data are expressed as mean ± SEM.

## Supporting Information

Data S1 (0.17 MB TIF)

## References

[1] Schon MP, Boehncke WH (2005). Medical progress - Psoriasis.. N Engl J Med.

[2] Bowcock AM, Krueger JG (2005). Getting under the skin: The immunogenetics of psoriasis.. Nat Rev Immunol.

[3] Lowes MA, Bowcock AM, Krueger JG (2007). Pathogenesis and therapy of psoriasis.. Nature.

[4] Sabat R, Philipp S, Hoeflich C, Kreutzer S, Wallace E (2007). Immunopathogenesis of psoriasis.. Exp Dermatol.

[5] Hancock GE, Kaplan G, Cohn ZA (1988). Keratinocyte growth regulation by the products of immune cells.. J Exp Med.

[6] Olaniran AK, Baker BS, Garioch JJ, Powles AV, Fry L (1995). A comparison of the stimulatory effects of cytokines on normal and psoriatic keratinocytes in vitro.. Arch Dermatol Res.

[7] Wolk K, Witte E, Philipp S, Asadullah K, Volk H (2007). A potential role of IL-20 in psoriasis vulgaris.. Exp Dermatol.

[8] Wolk K, Witte E, Wallace E, Doecke W, Kunz S (2006). IL-22 regulates the expression of genes responsible for antimicrobial defense, cellular differentiation, and mobility in keratinocytes: a potential role in psoriasis.. J Invest Dermatol.

[9] Albanesi C, De Pita O, Girolomoni G (2007). Resident skin cells in psoriasis: a special look at the pathogenetic functions of keratinocytes.. Clin Dermatol.

[10] Menon GK, Elias PM (1991). Ultrastructural-Localization of Calcium in Psoriatic and Normal Human Epidermis.. Arch Dermatol.

[11] Tu CL, Chang W, Bikle DD (2007). The role of the calcium sensing receptor in regulating intracellular calcium handling in human epidermal keratinocytes.. J Invest Dermatol.

[12] Tu CL, Oda Y, Komuves L, Bikle DD (2004). The role of the calcium-sensing receptor in epidermal differentiation.. Cell Calcium.

[13] Montell C (2006). TRP channels: Mediators of sensory signaling and roles in health and disease.. Chem Senses.

[14] Liao Y, Plummer NW, George MD, Abramowitz J, Zhu MX (2009). A role for Orai in TRPC-mediated Ca^2+^ entry suggests that a TRPC:Orai complex may mediate store and receptor operated Ca^2+^ entry.. Proc Natl Acad Sci U S A.

[15] Hofmann T, Obukhov AG, Schaefer M, Harteneck C, Gudermann T (1999). Direct activation of human TRPC6 and TRPC3 channels by diacylglycerol.. Nature.

[16] Beck B, V'yacheslav L, Roudbaraki M, Flourakis M, Charveron M (2008). TRPC channels determine human keratinocyte differentiation: New insight into basal cell carcinoma.. Cell Calcium.

[17] Beck B, Zholos A, Sydorenko V, Roudbaraki M, Lehen'kyi V (2006). TRPC7 is a receptor-operated DAG-activated channel in human keratinocytes.. J Invest Dermatol.

[18] Müller M, Hill K, Beschmann H, Rubant S, Boehncke WH (2008). Specific TRPC6 channel activation, a novel approach to stimulate keratinocyte differentiation.. J Biol Chem.

[19] Fatherazi S, Presland RB, Belton CM, Goodwin P, Al Qutub M (2007). Evidence that TRPC4 supports the calcium selective I-CRAC-like current in human gingival keratinocytes.. Pflügers Arch.

[20] Cai SW, Fatherazi S, Presland RB, Belton CM, Roberts FA (2006). Evidence that TRPC1 contributes to calcium-induced differentiation of human keratinocytes.. Pflügers Arch.

[21] Cai SW, Fatherazi S, Presland RB, Belton CM, Izutsu KT (2005). TRPC channel expression during calcium-induced differentiation of human gingival keratinocytes.. J Dermatol Sci.

[22] Leuner K, Kazanski V, Müller M, Essin K, Henke B (2007). Hyperforin a key constituent of St. John's wort specifically activates TRPC6 channels.. FASEB J.

[23] Pani B, Singh BB (2008). Darier's disease: a calcium-signaling perspective.. Cell Mol Life Sci.

[24] Woelfle U, Laszczyk MN, Kraus M, Leuner K, Kersten A (2010). Triterpenes Promote Keratinocyte Differentiation In Vitro, Ex Vivo and In Vivo: A Role for the Transient Receptor Potential Canonical (subtype) 6.. J Invest Dermatol.

[25] Jones KT, Sharpe GR (1994). Thapsigargin raises intracellular free calcium levels in human keratinocytes and inhibits the coordinated expression of differentiation markers.. Exp Cell Res.

[26] Gerdes J, Lemke H, Baisch H, Wacker HH, Schwab U (1984). Cell cycle analysis of a cell proliferation-associated human nuclear antigen defined by the monoclonal antibody Ki-67.. J Immunol.

[27] Karvonen SL, Korkiamaki T, Yla-Outinen H, Nissinen M, Teerikangas H (2000). Psoriasis and altered calcium metabolism: Downregulated capacitative calcium influx and defective calcium-mediated cell signaling in cultured psoriatic keratinocytes.. J Invest Dermatol.

[28] Tschachler E (2007). Psoriasis: the epidermal component.. Clin Dermatol.

[29] Wu Z, Hansmann B, Meyer-Hoffert U, Glaser R, Schroder JM (2009). Molecular identification and expression analysis of filaggrin-2, a member of the S100 fused-type protein family.. Plos One.

[30] Krueger GG, Chambers DA, Shelby J (1981). Involved and uninvolved skin from psoriatic subjects: are they equally diseased? Assessment by skin transplanted to congenitally athymic (nude) mice.. J Clin Invest.

[31] Leuner K, Heiser J, Derksen S, Mladenov M, Fehske CJ (2010). Simple 2,4 diacylphloroglucinols as TRPC6 activators – identification of a novel pharmacophore.. Mol Pharmacol.

[32] Rheinwald JG, Green H (1975). Formation of a keratinizing epithelium in culture by a cloned cell line derived from a teratoma.. Cell.

