# Supplementary figures and images for: Reduced TRPC Channel Expression in Psoriatic Keratinocytes Is Associated with Impaired Differentiation and Enhanced Proliferation
Source: PLoS One. 2011 Feb 22;6(2):e14716. doi: 10.1371/journal.pone.0014716 (PMC3043053; doi:10.1371/journal.pone.0014716)

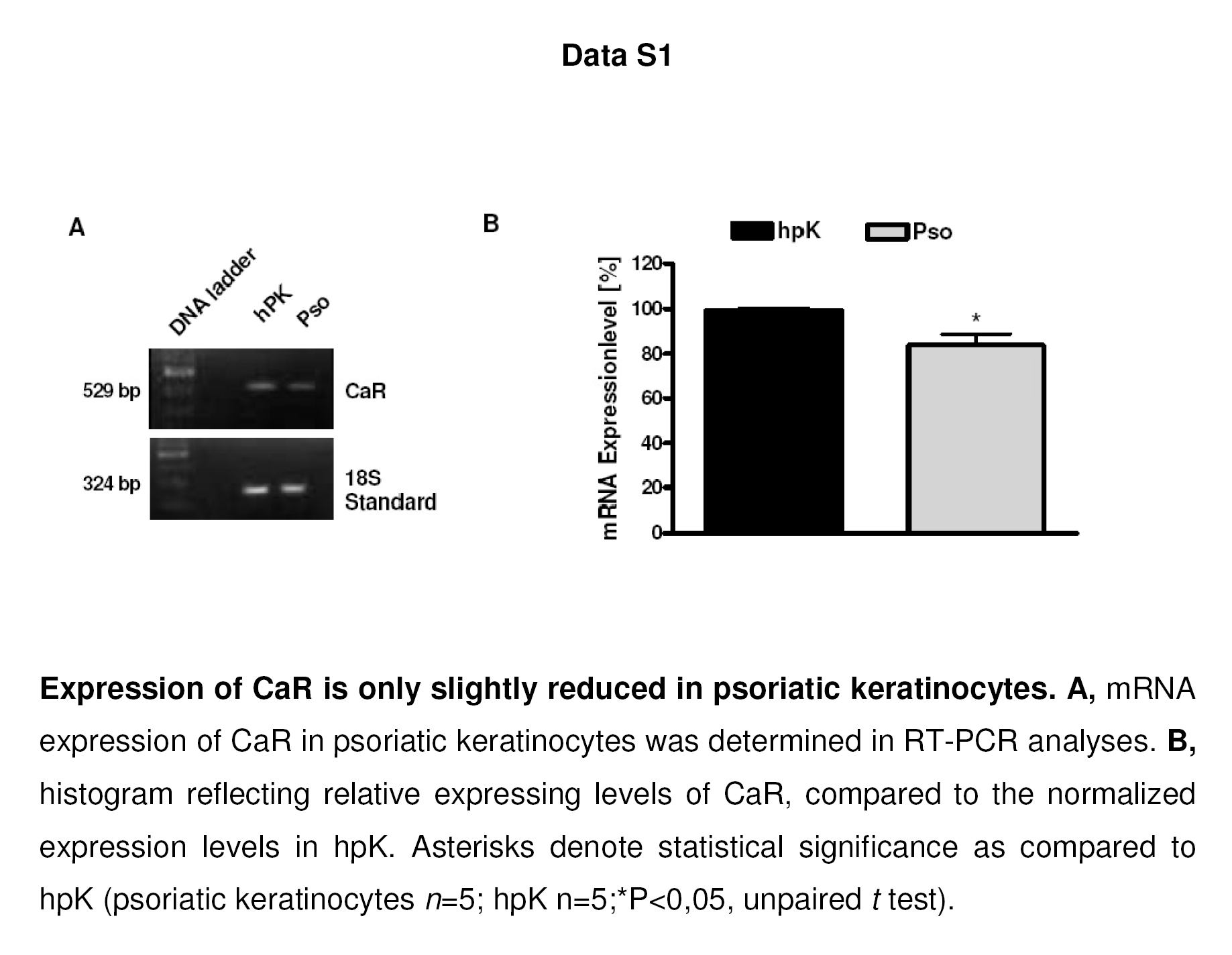

Supplement: Data S1 — (0.17 MB TIF) [file pone.0014716.s001.tif]
